# Micronutrients and Depression: The Role of Zinc, Magnesium and Selenium in Etiology, Mechanisms and Treatment

**DOI:** 10.1192/j.eurpsy.2026.11384

**Published:** 2026-07-31

**Authors:** M. F. Zeilstra, M. Arts, K. M. Zeilstra-Kalt, L. de Jonge

**Affiliations:** 1 Integrative Medicine, Center de Wetering, Leeuwarden; 2 Department of Geriatric Psychiatry and Neuropsychiatry, GGZ-WNB, Halsteren; 3 Integrative Medicine, Leonardo Scientific Research Institute, Groningen, Netherlands

## Abstract

**Introduction:**

Micronutrient deficiencies and depression are highly prevalent global health problems. While zinc, magnesium and selenium are essential for brain and immune functioning, their roles in the etiology and treatment of depression remain unclear.

**Objectives:**

This review aimed to (1) summarize empirical evidence on the associations between zinc, magnesium and selenium with depression; (2) discuss potential biological mechanisms; and (3) explore clinical implications for prevention and treatment.

**Methods:**

A narrative review was conducted, synthesizing animal, observational and intervention studies addressing serum levels, dietary intake or supplementation of zinc, magnesium and selenium in relation to depressive symptoms or diagnoses.

**Results:**

Zinc deficiency is consistently associated with increased risk of depression. Meta-analyses and clinical trials show lower zinc levels in depressed individuals and beneficial effects of zinc supplementation as an adjunct to antidepressants. Magnesium studies are mixed: cross-sectional evidence supports an inverse association with depression, while prospective and intervention studies report inconsistent findings. Selenium research is sparse and inconclusive; some studies suggest an optimal serum range, whereas others report null or paradoxical associations. Mechanistic pathways include modulation of neurotransmission (glutamatergic, serotonergic), HPA axis regulation, neurogenesis, oxidative stress, and inflammation.

**Conclusions:**

Evidence most strongly supports zinc as a relevant micronutrient in depression, with magnesium showing possible but inconsistent associations, and selenium requiring further study. Micronutrient deficiencies may increase vulnerability to depression through neurobiological and immune-inflammatory pathways. Future well-designed trials are warranted to clarify causal mechanisms and evaluate supplementation as an adjunctive treatment.

**Disclosure of Interest:**

None Declared

